# Nuclear receptors regulate lipid metabolism and oxidative stress markers in chondrocytes

**DOI:** 10.1007/s00109-016-1501-5

**Published:** 2017-01-09

**Authors:** Anusha Ratneswaran, Margaret Man-Ger Sun, Holly Dupuis, Cynthia Sawyez, Nica Borradaile, Frank Beier

**Affiliations:** 1 Department of Physiology and Pharmacology, Schulich School of Medicine & Dentistry, University of Western Ontario, London, ON N6A 5C1 Canada; 2 Western Bone & Joint Institute, University of Western Ontario, London, ON Canada

**Keywords:** Cartilage, Chondrocyte, Lipid metabolism, Osteoarthritis, Oxidative stress

## Abstract

**Abstract:**

Joint homeostasis failure can result in osteoarthritis (OA). Currently, there are no treatments to alter disease progression in OA, but targeting early changes in cellular behavior has great potential. Recent data show that nuclear receptors contribute to the pathogenesis of OA and could be viable therapeutic targets, but their molecular mechanisms in cartilage are incompletely understood. This study examines global changes in gene expression after treatment with agonists for four nuclear receptor implicated in OA (LXR, PPARδ, PPARγ, and RXR). Murine articular chondrocytes were treated with agonists for LXR, PPARδ, PPARγ, or RXR and underwent microarray, qPCR, and cellular lipid analyses to evaluate changes in gene expression and lipid profile. Immunohistochemistry was conducted to compare two differentially expressed targets (Txnip, Gsta4) in control and cartilage-specific PPARδ knockout mice subjected to surgical destabilization of the medial meniscus (DMM). Nuclear receptor agonists induced different gene expression profiles with many responses affecting lipid metabolism. LXR activation downregulated gene expression of proteases involved in OA, whereas RXR agonism decreased expression of ECM components and increased expression of *Mmp13.* Functional assays indicate increases in cell triglyceride accumulation after PPARγ, LXR, and RXR agonism but a decrease after PPARδ agonism. PPARδ and RXR downregulate the antioxidant Gsta4, and PPARδ upregulates Txnip. Wild-type, but not PPARδ-deficient mice, display increased staining for Txnip after DMM. Collectively, these data demonstrate that nuclear receptor activation in chondrocytes primarily affects lipid metabolism. In the case of PPARδ, this change might lead to increased oxidative stress, possibly contributing to OA-associated changes.

**Key message:**

- Nuclear receptors regulate metabolic genes in chondrocytes.
- Nuclear receptors affect triglyceride levels.
- PPARδ mediates regulation of oxidative stress markers.
- Nuclear receptors are promising therapeutic targets for osteoarthritis.

**Electronic supplementary material:**

The online version of this article (doi:10.1007/s00109-016-1501-5) contains supplementary material, which is available to authorized users.

## Introduction

Dysregulation of joint homeostasis can result in osteoarthritis (OA), a collective of heterogeneous pathologies culminating in joint failure. OA presents with similar pathological end points, but mechanisms of initiation and progression vary among subtypes of this disease, which is one of the leading causes of disability worldwide [1, 2]. Its varied presentation influences whether it is symptomatic or not and even whether it can be diagnosed radiographically. Multiple tissues, such as the articular cartilage, subchondral bone, synovium, meniscus, and fat pads, are involved in this condition, and initiation of this disease can stem from mechanical, metabolic, or age-associated factors, although none of these are mutually exclusive.

The main function of the cartilage is to act as a shock absorber, mediating load bearing through the influx and efflux of water attracted to the proteoglycan aggregates of the extracellular matrix and through the tensile strength conferred by collagen fibril organization [3]. Although cartilage cells contribute a small percentage of the volume of the entire joint, they are sensitive to external factors and respond with changes in gene expression affecting OA, thus underscoring their importance in joint homeostasis.

Metabolic OA has been classified as a distinct subtype of OA associated with disorders such as dyslipidemia, hypertension, and obesity [4]. Imbalances in systemic lipid and cholesterol metabolism, nutrient exchange, accumulation of advanced glycation end products, and increases in adipokines contribute to this condition. Changes in lipid metabolism, in particular, may directly affect joint homeostasis through ectopic lipid deposition in chondrocytes [4–6]. In fact, both chondrocyte-specific cholesterol accumulation and high-fat diet have caused increased disease severity in murine models [7–9]. Altogether, these data suggest direct regulation of cartilage homeostasis by lipid metabolism.

Nuclear receptors are a class of proteins that are activated by small molecule ligands and can up- or downregulate the expression of target genes through the recruitment of co-factors. They have been reported as attractive potential targets for pharmacological therapy because of their ability to bind synthetic or natural ligands that regulate transcriptional activity [10]. As such, synthetic agonists for nuclear receptors have been developed to target metabolic conditions such as dyslipidemia, atherosclerosis, and diabetes [11, 12]. Peroxisome proliferated activated receptors (PPARs) are typically involved in the control of lipid metabolism and activated by the binding of endogenous fatty acids, whereas liver X receptor (LXR) is principally involved in cholesterol metabolism. Recently, we have shown that cartilage-specific ablation of the gene encoding the nuclear receptor PPARδ has a protective effect on cartilage after surgical induction of OA, demonstrating that PPARδ promotes post-traumatic OA. Conversely, PPARγ and LXR are protective and necessary for normal joint function and skeletal development [13–16]. Interestingly, all three of these receptors act in heterodimers with the common partner RXR, positioning RXR at the center of a complex network of nuclear receptors. All of these proteins are expressed in the cartilage [15, 17, 18]. However, the mode of action of these proteins in the cartilage is largely unknown, and since they are transcription factors, identification of their target genes is essential to understand their specific roles and to evaluate their value as therapeutic targets. Here, we attempted to identify these target genes in a genome-wide manner.

In this study, we have used microarray analysis paired with functional validation to identify gene targets of LXR, PPARγ, PPARδ, and RXR in articular chondrocytes, in order to elucidate their potential role in OA pathogenesis. There is strong evidence implicating the involvement of nuclear receptors in the progression or prevention of OA, and here, we provide insight as to how they may be involved in altering the gene expression profile and phenotype of mature, healthy chondrocyte cultures. We are also the first, to our knowledge, to quantify changes in neutral lipid and free cholesterol mass in chondrocytes in vitro. This information is essential in uncovering the early changes that occur in chondrocytes before irreversible phenotypic changes within the joint and is vital since we currently have no effective biomarkers or treatment to alter the course of OA progression. Our data demonstrate that changes in gene regulation after nuclear receptor agonist treatment primarily affect lipid metabolism, suggesting a close link between lipid metabolism within chondrocytes and the progression of OA.

## Methods

### Primary cell culture and isolation

Immature murine articular chondrocytes (IMACs) were isolated from the femoral head, femoral condyle, and tibial condyles of 5–6-day-old CD1 mice (Charles River Laboratories) as per [19]. The tissue was then subjected to 1 h (3 mg/ml) followed by 24 h (0.5 mg/ml) incubations in Collagenase D diluted in Dulbecco’s Modified Eagles Medium supplemented with 2 mM *l*-glutamine, 50 U/ml penicillin, and 0.05 mg/ml streptomycin at 37 °C under 5% CO_2_. The tissue fragments were then agitated, and cells were isolated and cultured [19]. On the seventh day after isolation, cells were treated with either PPARδ agonist (GW501516), PPARγ agonist (Rosiglitazone), LXR agonist (GW3965), RXR agonist (SR11237), or control (DMSO), all at concentrations of 1 μM for 72 h. Pyruvate dehydrogenase kinase (PDK) inhibition studies were done using these nuclear agonist treatments and one of two inhibitors at 5 μM: dichloroacetate (DCA, pan-PDK inhibitor) or diisopropylaminedichloroacetate (DADA, PDK4 inhibitor), or vehicle control (water).

### RNA extraction, purification, and qPCR

Total RNA was isolated from cells using TRIzol® (Invitrogen). Cells were lysed in TRIzol® reagent, phases were separated using chloroform (20%), and supernatant was removed. RNA was precipitated using 100% isopropanol (0.5%) and washed using 70% ethanol followed by air-drying and resuspension in RNAse free water, as per manufacturer’s instructions. RNA was quantified using a Nanodrop 2000 spectrophotometer. RNA integrity was confirmed with Aligent 2100 BioAnalyzer Data Review Software (Wilmington, DE) at the London Regional Genomics Centre. Samples with RNA integrity number (RIN) values greater than 8 were used for microarray analysis.

Real-time PCR (qPCR) was performed as per [16]. In brief, qPCR was performed using a One-Step RT qPCR Master Mix kit and TaqMan Gene Expression Assays (Applied Biosystems), with 40 cycles on an ABI Prism 7900HT sequence detector (PerkinElmer), or on a Bio-Rad CFX384 RT-PCR system with 10–15 μl reaction volumes of iQ SYBR Green Supermix (Biorad) with diluted cDNA equivalent to 200–500 ng of input RNA per reaction, as well as 25–50 μM forward and reverse primers [20]. Probes for *Acan(*Mm00545794_m1), *Actb* (Mm02619580_g1), *Adamts4*(Mm00556068_m1), *Adamts5* (Mm00478620_m1), *Angptl4*(Mm00480431_m1), *Col2a1* (Mm01309565_m1), *Fabp3*(Mm02342495_m1), *Fabp4*(Mm00445878_m1), *LPL*(Mm00434764_m1), *Mmp2* (Mm00439498_m1), *Mmp3* (Mm00440295_m1), *Mmp13*(Mm00439491_m1), *Pdk4*(Mm01166879_m1), and *Sox9* (Mm00448840_m1) were purchased from Life Technologies. Gene expression was normalized relative to *Actb* or 18S (viability studies only). Relative gene expression was calculated using the ΔΔC_t_ method [21] as described [22]. Statistical analysis was conducted using GraphPad Prism 6.0. Values were transformed, and a one-way analysis of variance (ANOVA) was performed followed by Tukey’s multiple comparisons tests.

### Microarray and data analysis

Total RNA (200 ng per sample) was subject to 2 rounds of amplification followed by labeling and hybridization to Affymetrix GeneChip® Mouse Gene 2.0 ST Array containing 35,240 probes at the London Regional Genomics Centre (London, Ontario, Canada) as described [23]. Three independent cell and RNA isolations were used for each treatment. Probe data was analyzed, and gene level, ANOVA *p* values, and fold changes were determined using Partek Genomics Suite v6.6. Genes with at least 1.5-fold change, with *p* < 0.05 were considered significant and used for subsequent analyses. The complete array data set will be publicly available through Gene Expression Omnibus (GEO). The Venn diagrams were created using the online plotting tool Venny 2.0.1 [24]. KEGG pathway maps were generated using Ingenuity Pathway Analysis. Gene ontology biological processes and cellular component processes were classified through GO consortium available at geneontology.org using the PANTHER Overrepresentation Test (released 2016-07-15) and GO Ontology Database (released 2016-10-27), *Mus musculus* reference list, and Bonferroni correction for multiple testing. Biological processes identified with more than three genes involved were included in the table.

### Cellular lipid mass

IMACs were isolated, cultured, and treated with nuclear receptor agonists as described above. At the 72 h time point, cells were washed with 0.2% BSA in phosphate-buffered saline (PBS), followed by three washes in PBS. Lipids were extracted using 3:2 hexane/isopropanol solvent and pooled. Hexane/isopropanol solvents were evaporated to dryness under N_2_ and resuspended in 1.4 ml of chloroform–triton (0.5% triton *v*/*v*). Solvent was re-evaporated, and lipids were re-solubilized in 350 μl water. Two 50-μl aliquots were used per sample to determine total cholesterol (TC), free cholesterol (FC), and triglyceride (TG) mass, spectrophotometrically as per [25]. Cholesteryl esters (CE) were calculated by subtracting FC from TC. Proteins were extracted using 0.2 NaOH overnight incubation to digest chondrocytes and quantified using a standard BCA protein assay (Pierce, Thermo Fisher Scientific). All cell lipid measures reported are standardized to milligrams of cell protein. Values were normalized relative to vehicle control DMSO, and statistical analyses were performed using GraphPad Prism 6.0. Values were transformed, and a one-way analysis of variance (ANOVA) was performed followed by Tukey’s multiple comparisons tests.

### Animals and surgery

All animal experiments were approved by the Animal Use Subcommittee at The University of Western Ontario and were conducted in accordance with the guidelines from the Canadian Council on Animal Care. Mice were group housed (6 mice per cage) in colony cages on a standard 12 h light/dark cycle with free access to standard mouse chow, water, and running wheels. Surgical destabilization of the medial meniscus (DMM) or SHAM surgery was performed on 12-week-old C57BL/6 male mice (*N* = 8–9 per group), as described in [13]. Mice were euthanized at 10 or 12 weeks post-surgery for preparation of paraffin sections and subsequent histological analysis. Another cohort of 20-week-old male cartilage-specific *Ppard* knockout mice and wild-type littermate controls underwent DMM surgery (*N* = 5 per group) and was harvested for histological analyses 8 weeks later as in [13]. *Ppard* mutant mice were bred and genotypes as described in [13]. Paraffin sections from these studies were employed to evaluate the presence of Thioredoxin Interacting Protein (Txnip) and glutathione S-transferase A4 (Gsta4).

### Immunohistochemistry

Immunohistochemistry was performed on frontal sections of paraffin-embedded knee joints as described [26]. Txnip rabbit polyclonal antibody was purchased from Proteintech (18243-1-AP). Slides without primary antibody were used as controls, antigen retrieval was performed in 0.1% Triton in H_2_0, and primary antibody was used at a concentration of 1:300. Gsta4 rabbit polyclonal antibody was purchased from Proteintech (17271-1-AP), and immunohistochemical staining was performed under the same conditions as above, except with a primary antibody concentration of 1:100.

## Results

### Global changes in chondrocyte gene expression in response to nuclear receptor agonists

We have previously reported that treatment of articular chondrocytes with the PPARδ agonist GW501516 results in increased catabolic gene expression and robust fatty acid oxidation. We have also determined that the LXR agonist GW3965 delays chondrocyte hypertrophy [13, 16]. Identifying which genes are responsible for these phenotypes and how they interact with each other is key to understanding signaling pathways responsible for joint homeostasis and the prevention of osteoarthritis. We first examined global changes in chondrocyte gene expression in response to 1 μM treatment with LXR agonist GW3965, RXR agonist SR11237, PPARδ agonist GW501516, or PPARγ agonist Rosiglitazone. RNA was isolated from IMACs cultured with agonists for 72 h, then hybridized to Affymetrix microarrays representing the mouse genome.

**Fig. 1:**
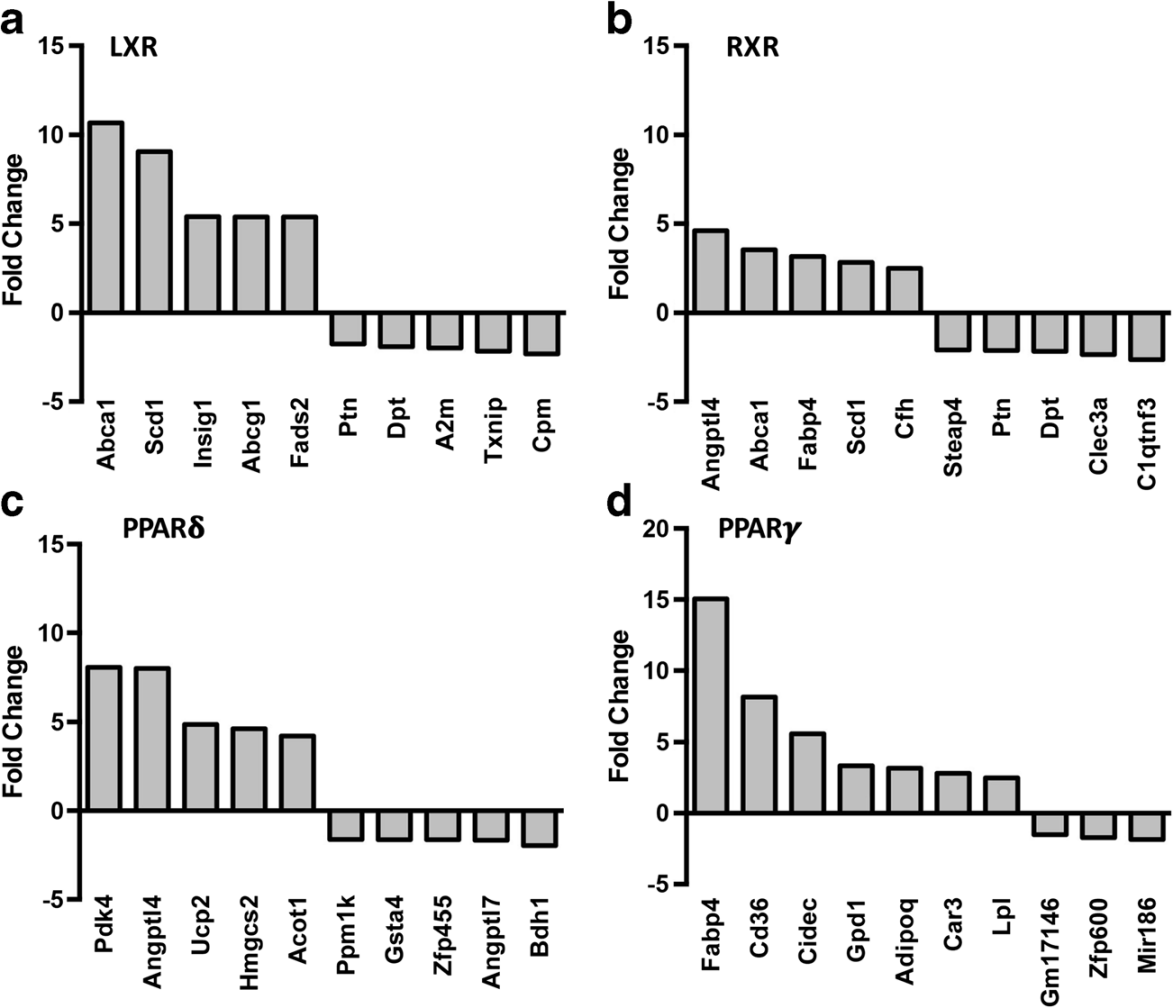
Microarray analyses of nuclear receptor agonist effects on chondrocyte gene expression. Microarray analysis of RNA isolated from immature murine articular chondrocytes treated for 72 h with 1 μM LXR agonist GW3965 (**a**), RXR agonist SR11237 (**b**), PPARδ agonist GW501516 (**c**), or PPARγ agonist Rosiglitazone (**d**). The most highly upregulated and downregulated genes are shown with fold change relative to vehicle control DMSO (1 μM)

We compiled a list of genes changed by more than 1.5-fold (refer to supplementary data for full list). LXR agonism significantly altered 128 genes (97 upregulated, 31 downregulated), RXR agonism differentially regulated a total of 108 genes (67 upregulated, 41 downregulated), PPARδ agonism induced changes in 58 genes (48 upregulated, 10 downregulated), while PPARγ agonism changed 32 genes (29 upregulated, 3 downregulated). The most robust and significantly upregulated and downregulated genes after nuclear receptor agonist treatment are shown in Fig. 1.

### Nuclear receptors affect common biological functions in chondrocytes

**Fig. 2:**
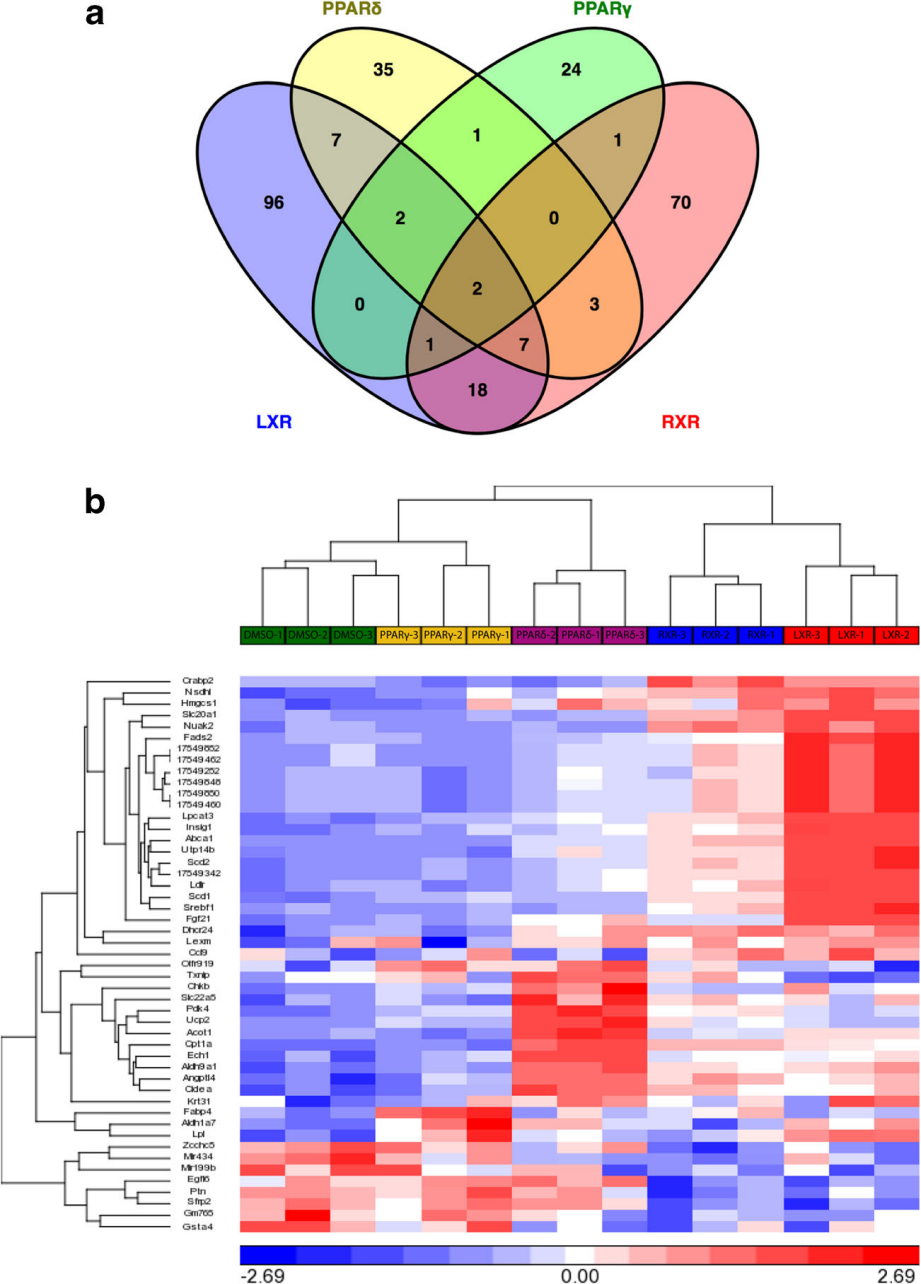
Comparison of nuclear receptor agonist effects on chondrocyte gene expression. **a** Comparison of all genes regulated by the four different nuclear receptor agonists on chondrocytes demonstrates that two genes are commonly regulated by all four nuclear receptors. Nine genes are commonly regulated by LXR, PPARδ, and RXR, while four genes are commonly regulated by LXR, PPARδ, and PPAR γ. Three genes are regulated by LXR, PPARγ, and RXR, and two genes are commonly regulated by PPARδ, PPARγ, and RXR. **b** Differences in regulation of genes commonly changed by all four nuclear receptor agonism are analyzed through hierarchical clustering and visually displayed in a heatmap

Comparison of gene expression profiles induced by various nuclear receptor agonists revealed several common hits. We therefore decided to evaluate shared functional roles by identifying similar biological processes through Gene Ontology. Supplementary Table 1 indicates the biological processes regulated by agonist treatment for each nuclear receptor, and common processes are highlighted with the same color. Both LXR and RXR agonism altered cholesterol biosynthetic processes, while LXR and PPARγ regulated triglyceride metabolism, and RXR and PPARγ increased metabolic processes in chondrocytes. We also investigated GO cellular component processes affected by each agonist (Supplementary Table 2). We noted that the extracellular matrix, mitochondrion, mitochondrial membrane, and endoplasmic reticulum were key areas in these processes, and that lipid particles and extracellular space were common between at least two treatment groups. The identified cellular components were consistent with our biological processes, heavily implicating the metabolic functions of these nuclear receptor agonists. In order to compare relationships between nuclear receptor agonist treatments, we created a Venn diagram to illustrate the number of genes induced by multiple receptors (Fig. 2a). The two genes upregulated by all four nuclear receptor agonists were *Pdk4* and *Angptl4*. Pdk4 functions as an inhibitor of the pyruvate dehydrogenase complex. Thus, it plays a key regulatory role in shifting energy utilization from glycolytic to fatty acid metabolism in the cell [27]. Angptl4 is a well-known direct target of PPARs that is upregulated by hypoxia and has been characterized as an adipocytokine [28]. It has also been identified as a potential pro-angiogenic mediator of arthritis, is involved in ECM remodeling, and is upregulated in the cartilage of RA and OA patients [29–31]. Larger numbers of genes were regulated by two or three different agonists (Fig. 2a), and differences in gene regulation between the commonly induced genes are hierarchically clustered and visually represented in a heatmap (Fig. 2b).

**Fig. 3:**
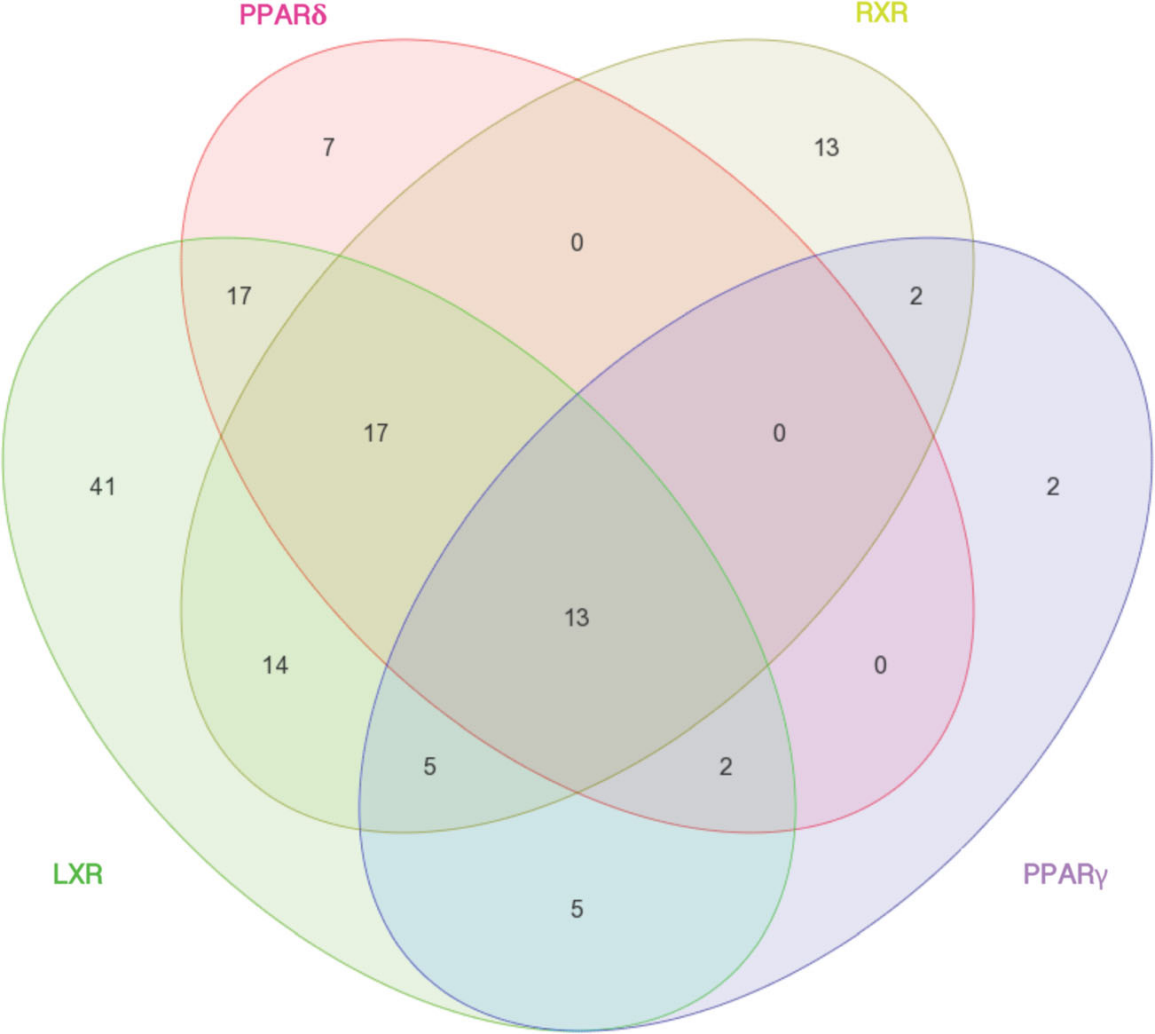
Comparison of nuclear receptor agonist effects on affected KEGG pathways. Comparison of all KEGG pathways enriched by the four different nuclear receptor agonist treatments in chondrocytes demonstrates that 13 pathways are commonly enriched by all four nuclear receptors. Seventeen pathways are commonly enriched by LXR, PPARδ, and RXR, while two pathways are commonly enriched by LXR, PPARδ, and PPARγ. Five pathways are commonly enriched by LXR, PPARγ, and RXR, and no pathways are commonly enriched by PPARδ, PPARγ, and RXR

KEGG pathway analysis was subsequently conducted to identify whether the genes common among agonist treatment would be associated with shared processes. We discovered that the four agonists shared 13 common pathways (Fig. 3). Of these, the most significantly enriched pathways included the PPAR signaling pathway and the adipocytokine signaling pathway (Supplementary Figs. 1 and 2). According to our analyses, all four nuclear receptors are involved in adipocyte differentiation and fatty acid transport. While LXR, PPARγ, and RXR regulate lipogenesis and LXR, PPARδ, and RXR mediate fatty acid oxidation and beta oxidation, only PPARδ agonism induces the ketogenic pathway.

Since PPARδ has opposite effects on OA progression than PPARγ and LXR, we were particularly interested in genes showing opposite responses to the respective agonists. However, the only gene that was differentially regulated (at our selected threshold) between any of the treatments was *Txnip*, which encodes the Thioredoxin interacting protein. Txnip inhibits Thioredoxin and contributes to ER stress, inflammasome activation, and the accumulation of reactive oxygen species (ROS) [32]. This gene was upregulated by PPARδ agonist GW501516 and downregulated by LXR agonist GW3965 treatment (Fig. 2b). Based on the common pathways and genes identified, we next validated changes in the expression of selected genes by qPCR.

### LXR, RXR, and PPAR agonism promote changes in genes involved in ECM homeostasis and chondrocyte metabolism

**Fig. 4:**
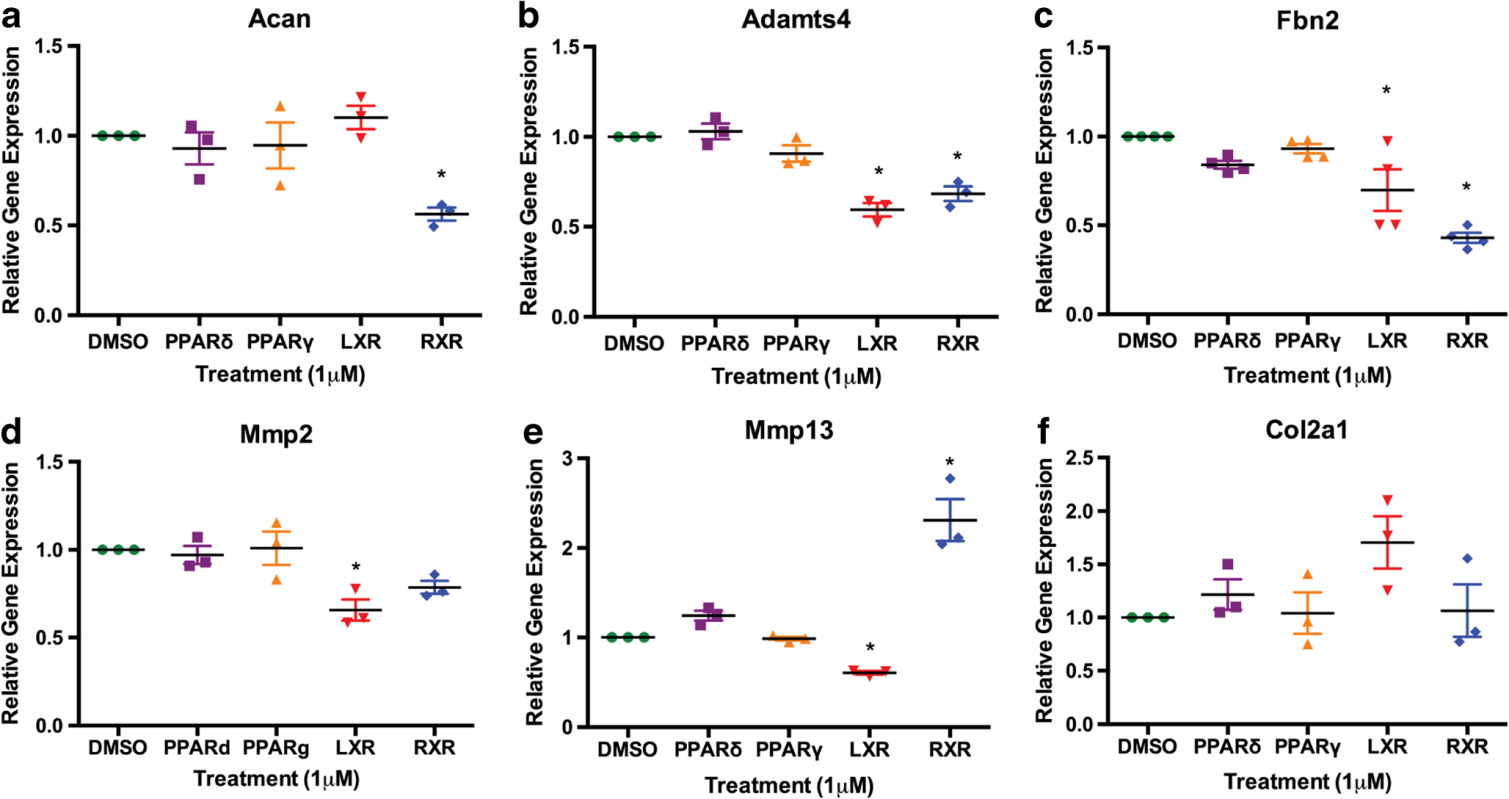
Effects of nuclear receptor agonist treatments on extracellular matrix gene expression in chondrocytes. IMACs were incubated for 72 h with 1 μM DMSO (vehicle control), PPARδ agonist GW501516, PPARγ agonist Rosiglitazone, LXR agonist GW3965, or RXR agonist SR11237. **a** Relative gene expression of *Acan* gene is significantly reduced by treatment with the RXR agonist. **b**, **c** Relative gene expression of *Adamts4* and *Fbn2* are significantly reduced by LXR and RXR agonist treatment. **d**, **e** Relative gene expression of matrix metalloproteinases *Mmp2* and *Mmp13* is decreased by LXR agonist treatment, while gene expression of *Mmp13* is significantly elevated by RXR agonist treatment. **f** *Col2a1* gene expression remains unchanged by all treatments. Values represented are the mean ± SEM of ≥3 independent cell isolations. *p < 0.05

Genes induced in the microarray were primarily involved in metabolic processes or in extracellular matrix component production and turnover. We chose to validate a subset of these genes that were shared between nuclear receptor agonist treatments. Aggrecan and Fibrillin 2 are extracellular matrix proteins encoded by the *Acan* and *Fbn2* genes. In concert with our microarray results, gene expression of *Acan* was significantly lower than vehicle control (DMSO) with RXR agonist treatment. Similarly, both LXR and RXR agonism significantly lowered gene expression of *Fbn2* (Fig. 4). Gene expression of Collagen 2 (*Col2a1)* remained unchanged in response to any of the treatments. Next, we validated expression of protease genes that were changed by some of the nuclear receptor agonists and accordingly found that gene expression of *Adamts4*, *Mmp2*, and *Mmp13* were significantly reduced by LXR agonism. Interestingly, RXR agonism decreased gene expression of *Adamts4* while increasing that of *Mmp13* (the primary collagenase of OA), implying a selective pathway for ECM remodeling and degradation.

**Fig. 5:**
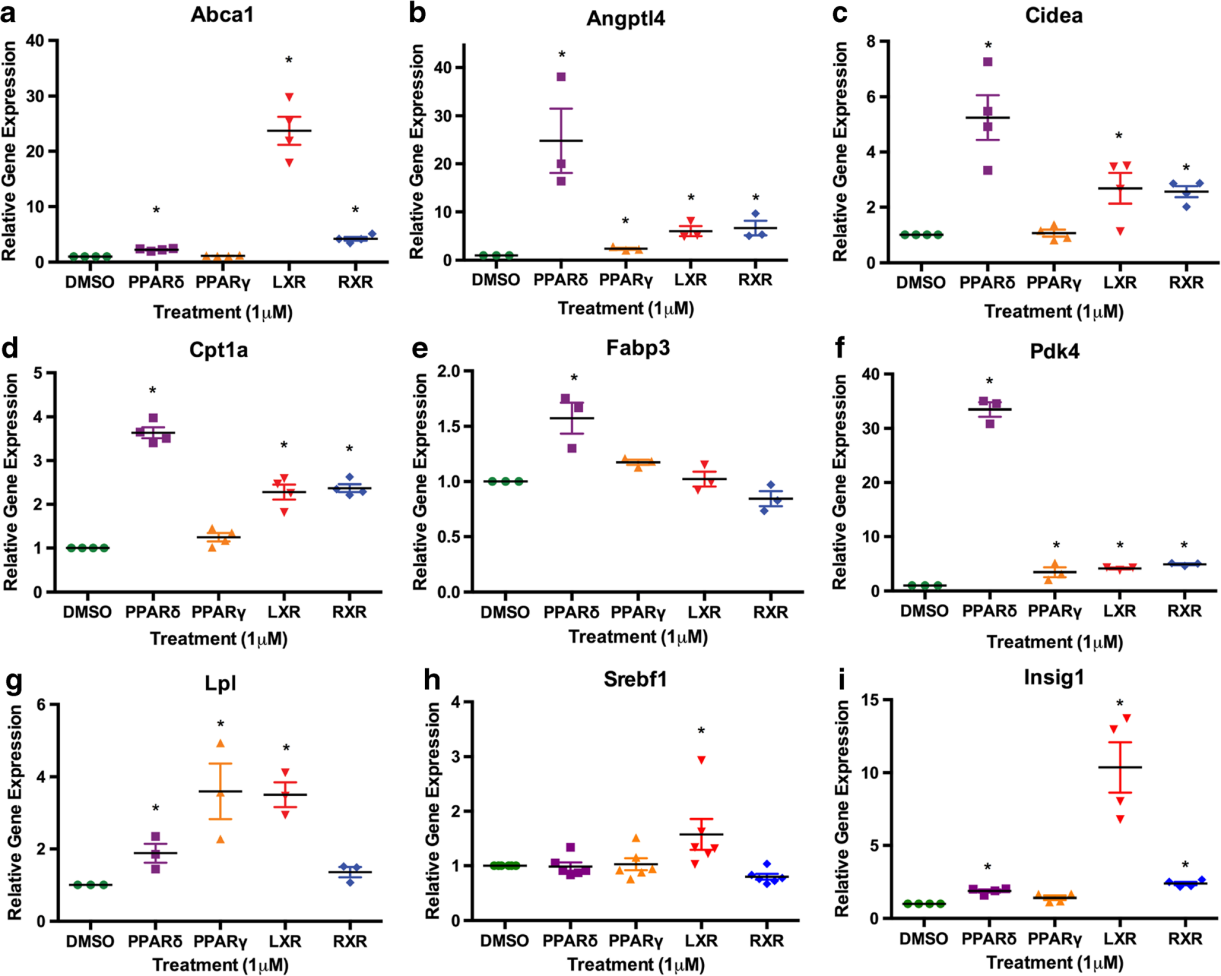
Effects of nuclear receptor agonist treatment on metabolic gene expression in chondrocytes. IMACs were incubated for 72 h with 1 μM DMSO (vehicle control), PPARδ agonist GW501516, PPARγ agonist Rosiglitazone, LXR agonist GW3965, or RXR agonist SR11237. **a**, **c**, **d**, **g**, **i** Relative gene expression of *Abca1, Cidea, Cpt1a, Lpl,* and *Insig1* is significantly increased by PPARδ, LXR, and RXR treatments. **b**, **f** Relative gene expression of *Angptl4* and *Pdk4* is elevated by all four treatments. **e** Relative gene expression of *Fabp3* is significantly upregulated by PPARδ agonism only. **g** RXR and PPARδ treatment significantly decreased relative gene expression of *Gsta4.* **h** *Srebf1* expression is significantly upregulated by LXR agonism only. Values represented are the mean ± SEM of ≥3 independent cell isolations. *p < 0.05

LXR, RXR, and PPARs are involved in the regulation of metabolism in a number of tissues. In a previous study, we showed that chondrocytes express functional PPARδ and are capable of responding to GW501516 stimulation with increased fatty acid oxidation [13]. All four nuclear receptor agonists induced strong effects on genes encoding metabolic enzymes. *Angptl4* and *Pdk4*, the two common genes induced by all four nuclear receptors in the microarray, demonstrated a similar robust upregulation in qPCR validation (Fig. 5). *Abca1*, *Cidea*, *Cpt1a*, *Lpl*, and *Insig1* were significantly increased by PPARδ, LXR, and RXR agonist treatment, and LXR also significantly increased the expression of *Srebf1.* Gene expression of cytoskeletal fatty acid transporter *Fabp3* was significantly increased by PPARδ activation, while *Gsta4*, a gene encoding an enzyme for cellular defense against reactive electrophiles [33], was significantly reduced by both PPARδ and RXR agonism.

In a previous study, our group has examined the toxicity of the administration of PPARδ agonist GW501516; we have shown that it does not alter cell number [13], while LXR stimulation increases cell number [16]. Here, we examined whether administration of any of the four nuclear receptor agonists affects cell physiology through changes in gene expression of markers for proliferation (*Ccnd1c, Pcna*), cell cycle exit (*p57/Cdkn1c*), apoptosis (*Bax*), or hypertrophic differentiation (*ColX, Runx2*) (Supplementary Fig. 3). We do not see any changes in these parameters, with the exception of RXR agonism decreasing the expression of *Runx2.*

### Increased expression of oxidative stress markers in osteoarthritic cartilage

**Fig. 6:**
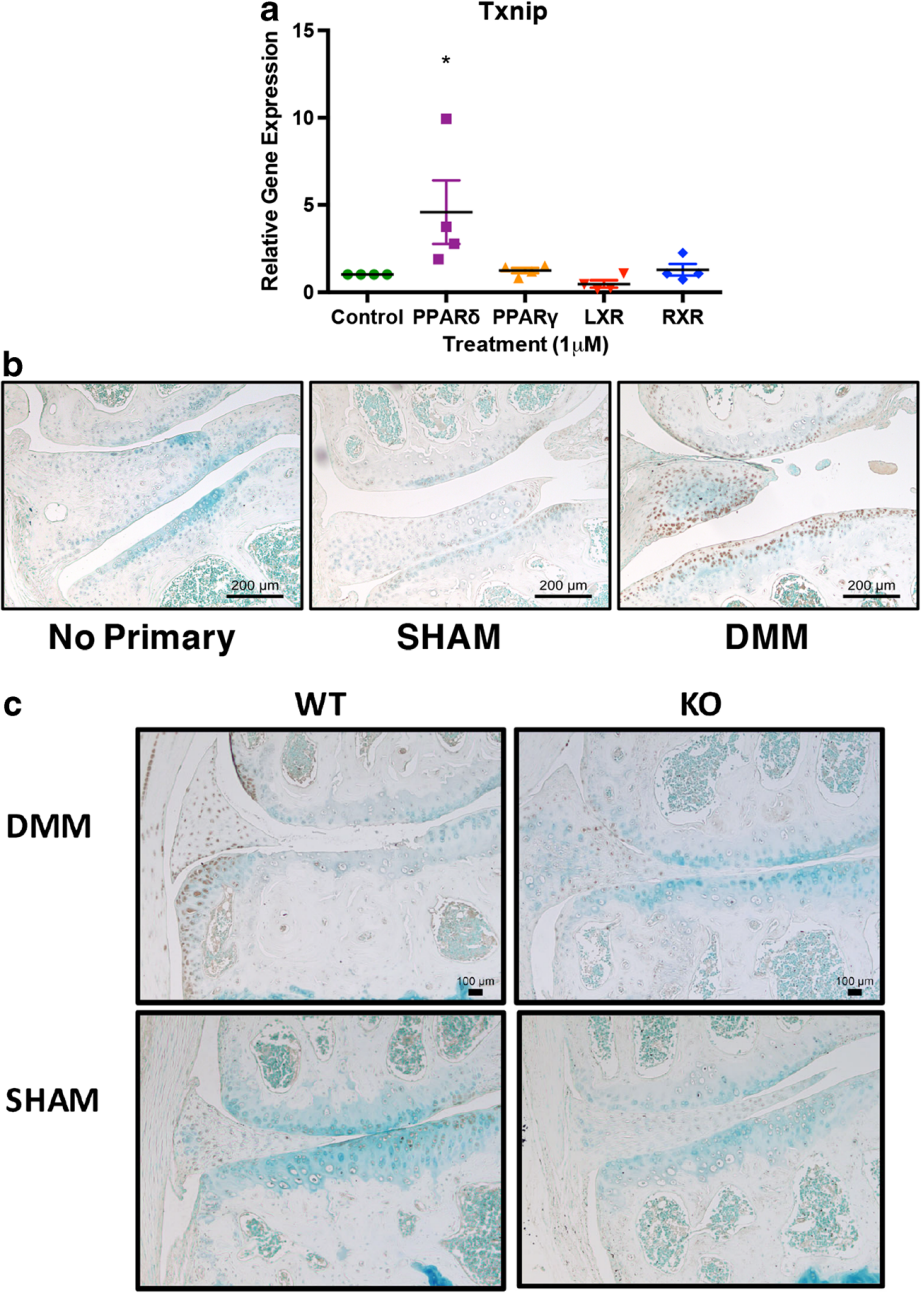
Effects of nuclear receptor agonist treatment on *Txnip* expression. **a** IMACs were incubated for 72 h with 1 μM DMSO (vehicle control), PPARδ agonist GW501516, PPARγ agonist Rosiglitazone, LXR agonist GW3965, or RXR agonist SR11237. PPARδ treatment significantly increased gene expression of *Txnip*. Values represented are the mean ± SEM of 4 independent cell isolations. *p < 0.05. **b** Immunohistochemistry for Txnip demonstrates increased cellular staining in the cartilage of WT DMM mice 10 weeks post-surgery relative to sham mice. **c** Immunohistochemical staining for Txnip in cartilage-specific *Ppard* KO mice vs WT littermate controls 8 weeks post DMM surgery. *Ppard* KO mice display less staining than WT mice

Txnip plays an important regulatory role in mediating oxidative stress and inflammation in a number of tissues [32]. *Txnip* was the only gene differentially regulated between nuclear receptor agonists. LXR agonist treatment downregulated gene expression, while PPARδ highly induced *Txnip*. These patterns observed in microarray analyses were evaluated by qPCR, where PPARδ agonism significantly increased gene expression of *Txnip*, while LXR agonist-treated cells demonstrated trends toward decreased gene expression, and RXR and PPARγ agonism showed no change (Fig. 6a). To examine whether Txnip expression is linked to OA, immunohistochemistry for Txnip was performed on frontal sections of mice after DMM surgery (Fig. 6b, c). Wild-type mice 10 weeks post-surgery had increased staining in remaining cartilage compared to mice that underwent sham surgery. To validate the effects of PPARδ on Txnip expression, we compared protein expression in cartilage-specific *Ppard* KO mice and wild-type littermates 8 weeks after DMM surgery. Wild-type mice demonstrated increased staining for Txnip after DMM surgery, particularly in areas of osteophyte growth at joint margins, whereas both sham-operated control mice and KO mice after either surgery showed little to no staining. The apparent increase of Txnip expression in the process of OA implies an imbalance in regulatory processes governing oxidative stress and inflammation, potentially linking changes in metabolism to osteoarthritic changes.

**Fig. 7:**
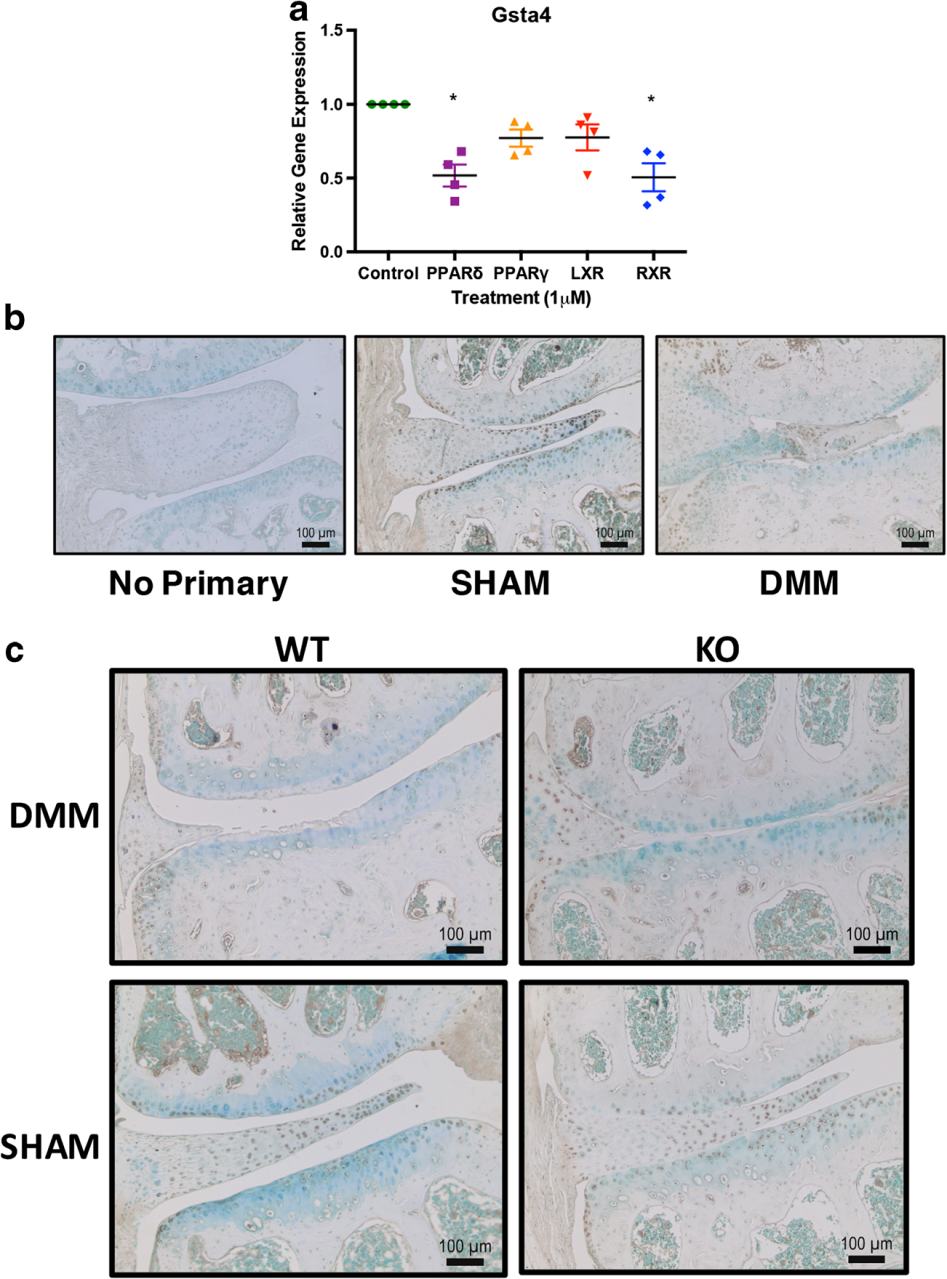
Effects of nuclear receptor agonist treatment on *Gsta4* expression. **a** IMACs were incubated for 72 h with 1 μM DMSO (vehicle control), PPARδ agonist GW501516, PPARγ agonist Rosiglitazone, LXR agonist GW3965, or RXR agonist SR11237. PPARδ agonist treatment and RXR agonist treatment both significantly decreased gene expression of *Gsta4*. Values represented are the mean ± SEM of 4 independent cell isolations. *p < 0.05. **b** Immunohistochemistry for Gsta4 demonstrates decreased cellular staining in the cartilage of WT DMM mice 12 weeks post-surgery relative to sham mice. **c** Immunohistochemical staining for Gsta4 in cartilage-specific *Ppard* KO mice vs WT littermate controls 8 weeks post-DMM or sham surgery. Both WT and *Ppard* KO mice display little to no staining of Gsta4 in the articular cartilage after DMM surgery, compared to sham controls.

In our qPCR validation of target genes, we saw decreased expression of *Gsta4 (*Fig. 7a) after PPARδ and RXR agonist treatment. Gsta4 protects the cell from reactive aldehydes that are produced as a result of lipid peroxidation or oxidative stress. We assessed the localization of Gsta4 in the DMM model and found that sham-operated animals displayed consistent immunohistochemical staining in superficial cartilage and meniscus, both in wild-type and cartilage-specific *Ppard* KO mice (Fig. 7b, c). DMM-operated animals showed little to no staining in cartilage of both genotypes, even if superficial cartilage remained intact.

### Changes in gene expression correspond with functional changes in chondrocyte lipid profile

**Fig. 8:**
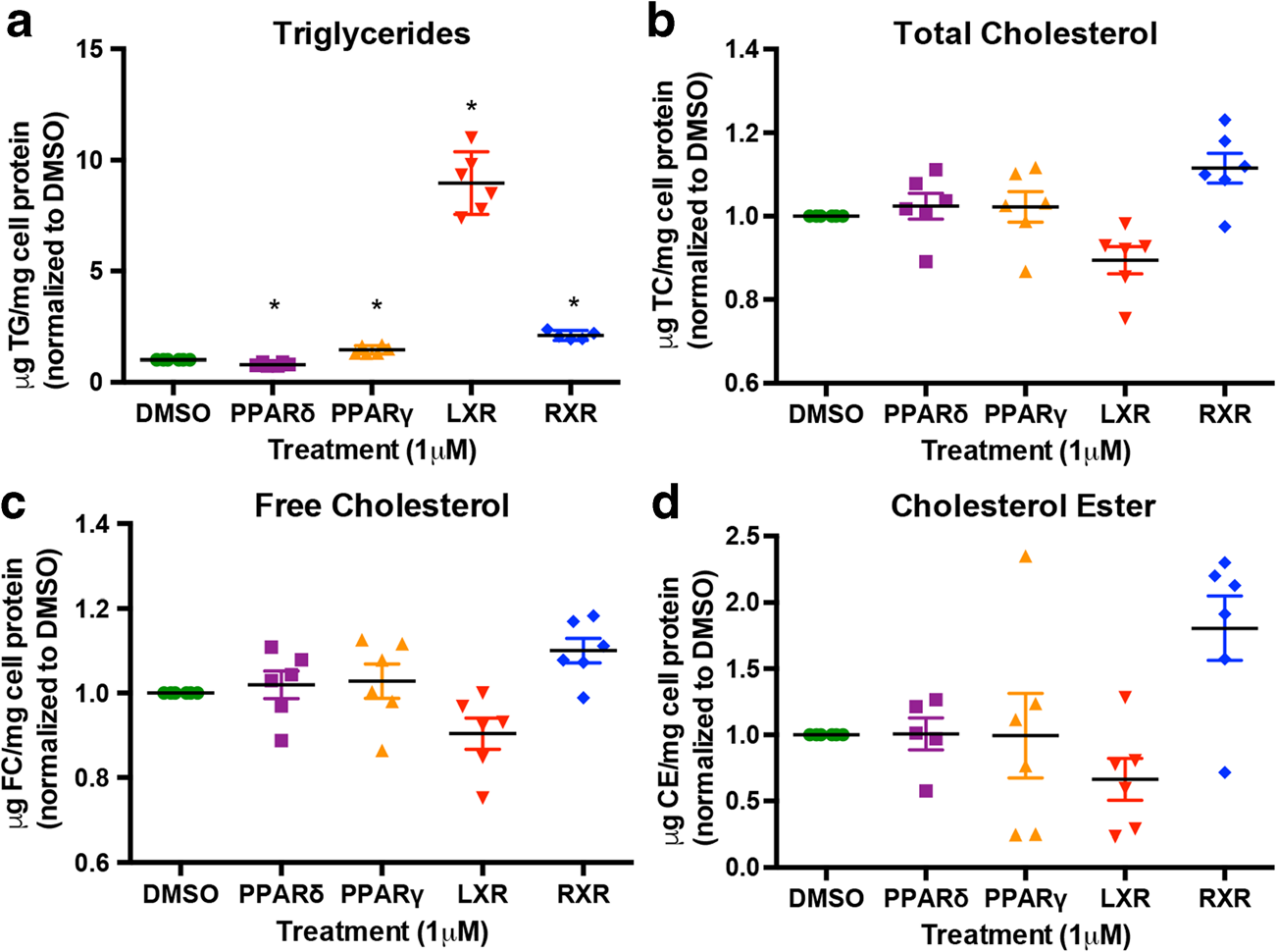
Quantification of cellular lipid mass in nuclear receptor agonist-treated chondrocytes. IMACs were incubated for 72 h with 1 μM DMSO (vehicle control), PPARδ agonist GW501516, PPARγ agonist Rosiglitazone, LXR agonist GW3965, or RXR agonist SR11237. Lipids were extracted, isolated, and mass was measured spectrophotometrically. Proteins were isolated and quantified using BCA. Measurements are reported relative to mg of cell protein. Absolute mean values for triglycerides, total cholesterol, free cholesterol, and cholesterol esters were 7.4, 27.1, 26.4, and 0.75 μg/mg protein, respectively, for DMSO-treated cells. **a** Cell triglycerides (μg) are significantly elevated by PPARγ, LXR, and RXR treatment and are significantly decreased by PPARδ agonism. **b**, **c**, **d** Total cholesterol, free cholesterol, and cholesterol ester remain unchanged after nuclear receptor agonist treatment. Values represented are the mean ± SEM of ≥5 independent cell isolations. *p < 0.05

In light of the number of genes involved in lipid metabolism that were identified in our gene expression analyses, we assessed neutral lipid and cholesterol accumulation in chondrocytes. Using the same nuclear receptor agonist treatment protocols, we harvested IMACs for cellular lipid mass assays. These assays allowed us to directly quantify triglycerides and cholesterol in vitro. There were significant changes in cell triglycerides, but not total cholesterol, free cholesterol, or cholesteryl esters (Fig. 8). These data suggest that changes in lipid metabolism upon agonist treatment are likely related to lipogenesis and fatty acid oxidation, rather than cholesterol transport or accumulation. In particular, triglycerides were significantly decreased with PPARδ agonist treatment and were significantly elevated with LXR, PPARγ, and RXR agonism. These changes are consistent with the known effects of activation of these nuclear receptors on triglyceride metabolism in other cell types and suggest that PPARδ may have an opposing role in lipid metabolism in chondrocytes relative to the other nuclear receptors examined [34].

We investigated whether altering metabolic pathways would affect lipid metabolism in chondrocytes treated with the nuclear receptor agonists. Given the effects of all agonists on *Pdk4*, we performed inhibition of pyruvate dehydrogenase kinase with a pan-PDK inhibitor (DCA) or PDK4 specific inhibitor (DADA) (Supplementary Fig. 4). PDK enzymes act to inhibit pyruvate dehydrogenase which catalyzes the first step of the pyruvate dehydrogenase complex (PDC). The PDC oxidizes pyruvate to generate acetyl-coA to be used in the TCA cycle, thereby promoting the preferential oxidation of glucose. Either treatment did not affect the responses of chondrocyte triglyceride levels to the four agonists.

## Discussion

This study is among the first to examine changes in global gene expression in chondrocytes after nuclear receptor agonist treatment, particularly paired with concurrent functional analysis. It provides compelling evidence that nuclear receptors drive early changes in cell metabolism that can influence deleterious changes in cellular phenotype leading to the progression of OA. Nuclear receptors have been increasingly linked to the progression of OA. We have previously established the degenerative changes promoted by PPARδ agonism in cartilage, as well as the beneficial and necessary role of PPARγ in cartilage [13, 14]. We and others have characterized the protective role of LXR in osteoarthritis [16, 35, 36]. However, in order to establish how or whether these ligand-activated receptors are feasible therapeutic targets, we must examine the molecular changes linked to activation or inhibition of each factor.

We used IMACs treated with LXR, RXR, PPARγ, or PPARδ agonists for 72 h. Immature murine articular chondrocytes provide a large number of cells for analyses on fully differentiated primary chondrocytes while minimizing dedifferentiation [19]. Microarray analyses of IMACs revealed changes in metabolic and ECM genes in response to these agonists; these changes were largely confirmed by qPCR. Agonism of RXR decreased gene expression of the major ECM component aggrecan and increased the expression of ECM protease *Mmp13*, while LXR agonism decreased the gene expression of proteases *Adamts4*, *Mmp2*, and *Mmp13*. Of particular interest were the increases in expression of genes involved in lipid metabolism since they showed greater induction than those regulating ECM turnover. Among these genes, two were induced by all four agonists, *Pdk4* and *Angptl4*, suggesting that they might play central roles in cartilage metabolism. Interestingly, in an earlier study, we had also demonstrated increased expression of *Pdk4* in response to dexamethasone, a ligand for the glucocorticoid receptor which is another member of the nuclear receptor family [37].

Functional evaluation of lipid metabolism using cellular lipid mass assays demonstrated a significant decrease in triglycerides after PPARδ agonist treatment. Conversely, triglycerides were significantly increased with PPARγ, LXR, and RXR agonists. This is not surprising as PPARγ can often act antagonistically to PPARδ with regard to lipogenesis [38], while LXR mediates fatty acid biosynthesis through activation of genes such as *Srebf1*, *Fasn*, and *Scd1* which corroborates our data (see GEO dataset) [39, 40]. Quantification of cell lipids in vitro enables us to assess differences in some aspects of lipid metabolism between treatments. In fact, it is plausible that the dysregulation in lipid metabolism that we observed could initiate metabolic changes in the cell that eventually lead to apoptosis, inflammation, or changes in cell behavior, such as synthesis of catabolic factors. Increased lipid deposition in osteoarthritic cartilage has been shown, while increased reactive oxygen species (ROS) can cause lipid peroxidation, which in turn could cause oxidative stress, resulting in degenerative changes to the matrix through oxidation of collagen II [6]. We also sought to reduce the dysregulation of cell lipids through the pharmacological inhibition of PDK, which would increase ATP synthesis through glucose oxidation [41]. However, we found that further attempting to shift the preferred metabolic pathway away from fatty acid oxidation by promoting an alternate pathway did not change cellular triglyceride levels and agonist responses.

In addition to dysregulation of cell lipids, we also see significantly decreased *Gsta4* expression after PPARδ or RXR agonist treatment. The encoded enzyme Glutathione S-transferase A 4 protects against HNE (4 Hydroxynonal)-induced damage in chondrocytes. HNE is an extremely reactive aldehyde produced from ROS and lipid peroxidation and is increased in synovial fluid from OA patients [40] as well as articular cartilage [42]. HNE can also post-transcriptionally modify Collagen 2 and MMP13 to induce degradative changes in cartilage as well as changes in cell–matrix interactions [43, 44]. Molecularly, it has been observed that HNE can stimulate COX-2 via the ATF/cAMP response element and inhibits iNOS and NF-kB inactivation in human articular chondrocytes [45]. GSTs are major determinants of intracellular HNE concentration, and dysregulation in disease states can result in toxic effects [46]. The GSTA4 isoform has well-established selectivity and efficiency for conjugation of lipid peroxidation products, especially HNE [46, 47]. Accordingly, decreased levels of GSTA4 are present in human OA cartilage, making the cartilage more susceptible to damage [33]. In addition, IL-1β treatment of human articular chondrocytes decreases expression of *GSTA4*, while inhibition of the p38 MAPK pathway (an important signaling pathway in the pathogenesis of OA) increases levels of *GSTA4* [48]*.* This suggests that the catabolic effects of PPARδ (and possibly RXR) in cartilage could be partially due to the decreased expression of *Gsta4*, resulting in decreased anti-oxidant defense and thus increased oxidative stress. In our surgical model of DMM induction, we see that even the minimal damage experienced by PPARδ KO after surgery is enough to decrease the expression of this protein in vivo, indicating that *Gsta4* is an early response gene to damage and that there may be multiple pathways that influence its regulation.

Another gene of interest, *Txnip* (thioredoxin interacting protein), was highly induced by PPARδ agonism, but appeared repressed by LXR agonism, in agreement with the opposing effects of these nuclear receptors on OA progression. Thioredoxin is another important antioxidant, but binding of Txnip to thioredoxin inhibits its ability to scavenge for ROS [49]. In humans, decreased *TXNIP* is seen at late stages of OA, while increased *TXNIP* is seen in H_2_O_2_-induced cartilage damage in human chondrocytes [50, 51]. In our study, we demonstrate increased gene expression of *Txnip* after PPARδ agonism in chondrocytes, and control mice show increased staining for Txnip after DMM surgery. The apparent differences in Txnip regulation in human and mouse OA are likely due to the different disease stages; human data were obtained from end-stage samples, while the DMM model is subtle and our analyses have largely focused on mild to moderate disease. We also show that cartilage-specific *Ppard* knockout mice that are protected against cartilage degeneration in the DMM model have decreased Txnip staining after DMM surgery, in line with our microarray data. Txnip has been linked to oxidative stress and inflammation, and it can directly activate NF-kB and downstream inflammatory cytokines [52]. In chondrocytes, recent work has shown that Redd1 can form a complex with Txnip to regulate autophagy [53]. Taken altogether, these data help to form a cohesive picture of how changes in cell metabolism could influence the development of early osteoarthritis. Nuclear receptors appear to play a key role in these processes by regulating the expression of central players such as *Txnip* and *Gsta4*.

Current treatment strategies for OA are largely ineffective or inconclusive. It is possible that we are missing a critical temporal period during which chondrocyte homeostasis is disrupted, later leading to matrix degeneration. Recent evidence demonstrates that nuclear receptors are key regulators of OA pathogenesis, and the data presented here suggests that their primary targets are metabolic regulation. Metabolic deregulation, in turn, can trigger events leading to oxidative stress and inflammation, protease activation, and ultimately cartilage degeneration. Targeting these critical processes could be a promising avenue for treatments that alter disease progression.

## Electronic supplementary material

ESM 1 (PDF 3578 kb).
