# Supplementary material for: Nuclear receptors regulate lipid metabolism and oxidative stress markers in chondrocytes
Source: J Mol Med (Berl). 2017 Jan 9;95(4):431–44. doi: 10.1007/s00109-016-1501-5 (PMC5357281; doi:10.1007/s00109-016-1501-5)
Supplement: Supplementary file 1 — (PDF 3578 kb) [file 109_2016_1501_MOESM1_ESM.pdf]

**Electronic supplementary material (ESM)**

*J Mol Med 2016*

# Nuclear receptors regulate lipid metabolism and oxidative stress markers in chondrocytes

Anusha Ratneswaran[1,2*], Margaret Man-Ger Sun[1,2*], Holly Dupuis[1,2], Cynthia Sawyez[1], Nica Borradaile[1], and Frank Beier[1,2, #]

* Contributed equally

[1] Department of Physiology and Pharmacology, Schulich School of Medicine & Dentistry, University of Western Ontario, London, ON, Canada

[2] Western Bone & Joint Institute, University of Western Ontario, London, ON, Canada

# To whom correspondence should be sent: Dr. Frank Beier

Department of Physiology and Pharmacology, University of Western Ontario, London, ON, Canada; London, N6A 5C1; phone: 519-661-2111 ext 85344; email: fbeier@uwo.ca

| GO Biological Process | # of Genes Involved | Fold Enrichment |
|---|---|---|
| **PPARγ** | | |
| regulation of sequestering of triglyceride (GO:0010889) | 3 | > 100 |
| lipid particle organization (GO:0034389) | 3 | > 100 |
| regulation of macrophage derived foam cell differentiation (GO:0010743) | 3 | > 100 |
| positive regulation of lipid storage (GO:0010884) | 3 | > 100 |
| regulation of lipid storage (GO:0010883) | 4 | 92.81 |
| triglyceride metabolic process (GO:0006641) | 5 | 75.3 |
| fat cell differentiation (GO:0045444) | 4 | 33.67 |
| positive regulation of lipid metabolic process (GO:0045834) | 5 | 31.33 |
| chemical homeostasis (GO:0048878) | 8 | 8.31 |
| **RXR** | | |
| cholesterol biosynthetic process (GO:0006695) | 5 | 42.29 |
| positive regulation of lipid metabolic process (GO:0045834) | 7 | 12.53 |
| anion transport (GO:0006820) | 11 | 6.35 |
| regulation of phosphorus metabolic process (GO:0051174) | 20 | 3.07 |
| negative regulation of biological process (GO:0048519) | 38 | 2.12 |
| **PPARδ** | | |
| carnitine metabolic process (GO:0009437) | 4 | > 100 |
| long-chain fatty acid metabolic process (GO:0001676) | 5 | 23.53 |
| alcohol metabolic process (GO:0006066) | 7 | 12.16 |
| regulation of lipid metabolic process (GO:0019216) | 8 | 11.95 |
| small molecule biosynthetic process (GO:0044283) | 9 | 10.82 |
| lipid biosynthetic process (GO:0008610) | 9 | 9.63 |
| **LXR** | | |
| cholesterol biosynthetic process (GO:0006695) | 12 | 82.46 |
| regulation of cholesterol biosynthetic process (GO:0045540) | 4 | 49.82 |
| acetyl-CoA metabolic process (GO:0006084) | 5 | 39.86 |
| isoprenoid biosynthetic process (GO:0008299) | 5 | 33.21 |
| response to fatty acid (GO:0070542) | 6 | 29.16 |
| regulation of plasma lipoprotein particle levels (GO:0097006) | 6 | 28.47 |
| triglyceride metabolic process (GO:0006641) | 7 | 24.47 |
| fatty acid biosynthetic process (GO:0006633) | 13 | 23.99 |
| regulation of fatty acid metabolic process (GO:0019217) | 7 | 18.6 |
| positive regulation of lipid biosynthetic process (GO:0046889) | 7 | 18.12 |
| cellular response to starvation (GO:0009267) | 9 | 15.73 |
| lipid homeostasis (GO:0055088) | 7 | 12.24 |
| lipid localization (GO:0010876) | 9 | 7.57 |
| oxidation-reduction process (GO:0055114) | 21 | 4.85 |

**Table 1:** Biological processes differentially regulated by nuclear receptor agonists as indicated by Gene Ontology (GO) bioinformatics analysis. Commonly regulated biological processes between treatments are indicated by the same color.

| GO Cellular Component | # of Genes Involved | Fold Enrichment |
|---|---|---|
| **PPARγ** | | |
| lipid particle (GO:0005811) | 4 | 50.62 |
| **RXR** | | |
| extracellular matrix component (GO:0044420) | 6 | 10.99 |
| proteinaceous extracellular matrix (GO:0005578) | 15 | 10.59 |
| extracellular space (GO:0005615) | 19 | 3.18 |
| **PPARδ** | | |
| mitochondrial membrane (GO:0031966) | 9 | 6.87 |
| **LXR** | | |
| lipid particle (GO:0005811) | 5 | 13.63 |
| integral component of endoplasmic reticulum membrane (GO:0030176) | 7 | 10.27 |
| peroxisome (GO:0005777) | 8 | 9.67 |
| extracellular space (GO:0005615) | 22 | 2.63 |
| mitochondrion (GO:0005739) | 26 | 2.6 |
| extracellular exosome (GO:0070062) | 32 | 2.16 |

**Table 2:** Cellular components differentially regulated by nuclear receptor agonists as indicated by Gene Ontology bioinformatics analysis. Commonly regulated cellular components between treatments are indicated by the same color.

| Pathway Name | Enrichment P-Value | | | |
|---|---|---|---|---|
| | PPARδ | LXR | PPARγ | RXR |
| PPAR signaling pathway* | 7.91E-07 | 2.33E-11 | 5.30E-12 | 1.56E-07 |
| Fatty acid metabolism | 4.86E-05 | 5.14E-14 | 0.0062512 | 5.26E-06 |
| Adipocytokine signaling pathway* | 0.0337 | 0.0351 | 5.78E-04 | 0.00563 |
| Biosynthesis of unsaturated fatty acids | 0.00469 | 3.83E-09 | 0.0585 | 2.78E-04 |
| Fatty acid degradation | 1.46E-06 | 1.35E-04 | 0.112 | 0.0267 |
| Metabolic pathways | 9.78E-07 | 2.74E-08 | 0.157 | 0.456 |
| Peroxisome | 0.0449 | 0.00149 | 0.0162 | 0.35 |
| Glycerophospholipid metabolism | 0.052 | 0.0634 | 0.0189 | 0.374 |
| Fat digestion and absorption | 0.146 | 0.326 | 0.00365 | 0.18 |
| Ovarian steroidogenesis | 0.215 | 0.452 | 0.131 | 0.0364 |
| Retinol metabolism | 0.052 | 0.604 | 0.194 | 0.374 |
| Olfactory transduction | 0.95 | 0.9999 | 0.755 | 0.833 |
| Alzheimer's disease | 0.531 | 0.848 | 0.355 | 0.614 |

**Table 3:** KEGG pathways commonly affected by all four nuclear receptor agonist treatments with corresponding enrichment p-values. Extensive pathway maps are provided in subsequent supplementary figures for pathways marked with asterisks (*).

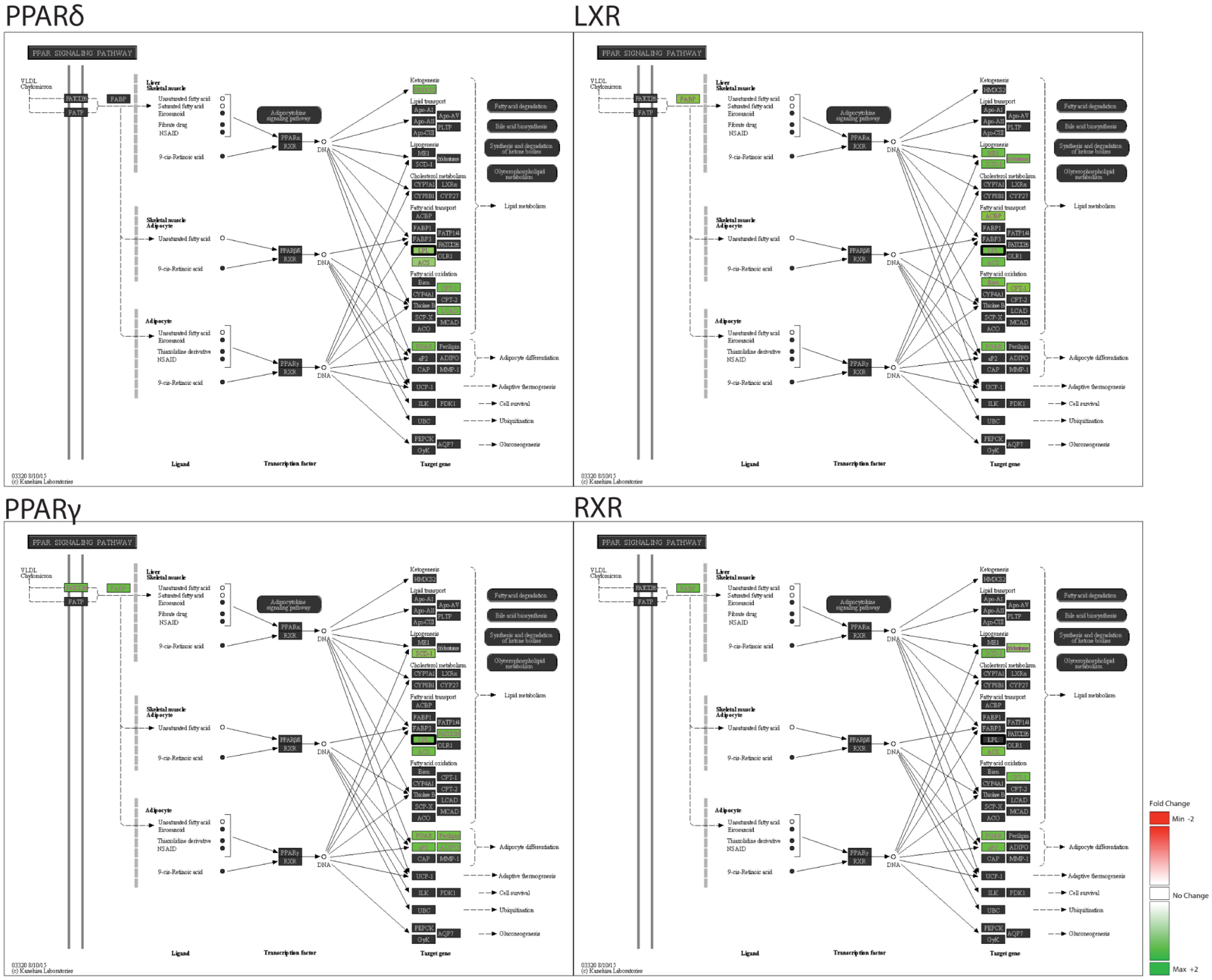


**Supplementary Figure 1: Pathway mapping of PPAR signaling.** The PPAR signaling pathway is significantly enriched by all four nuclear receptor agonist treatments. Differentially expressed genes as a resulted of each nuclear receptor agonism is mapped onto the PPAR signaling pathway, with gene expression fold-changes ranging from -2 to +2 represented by a color gradient of red or green, respectively.

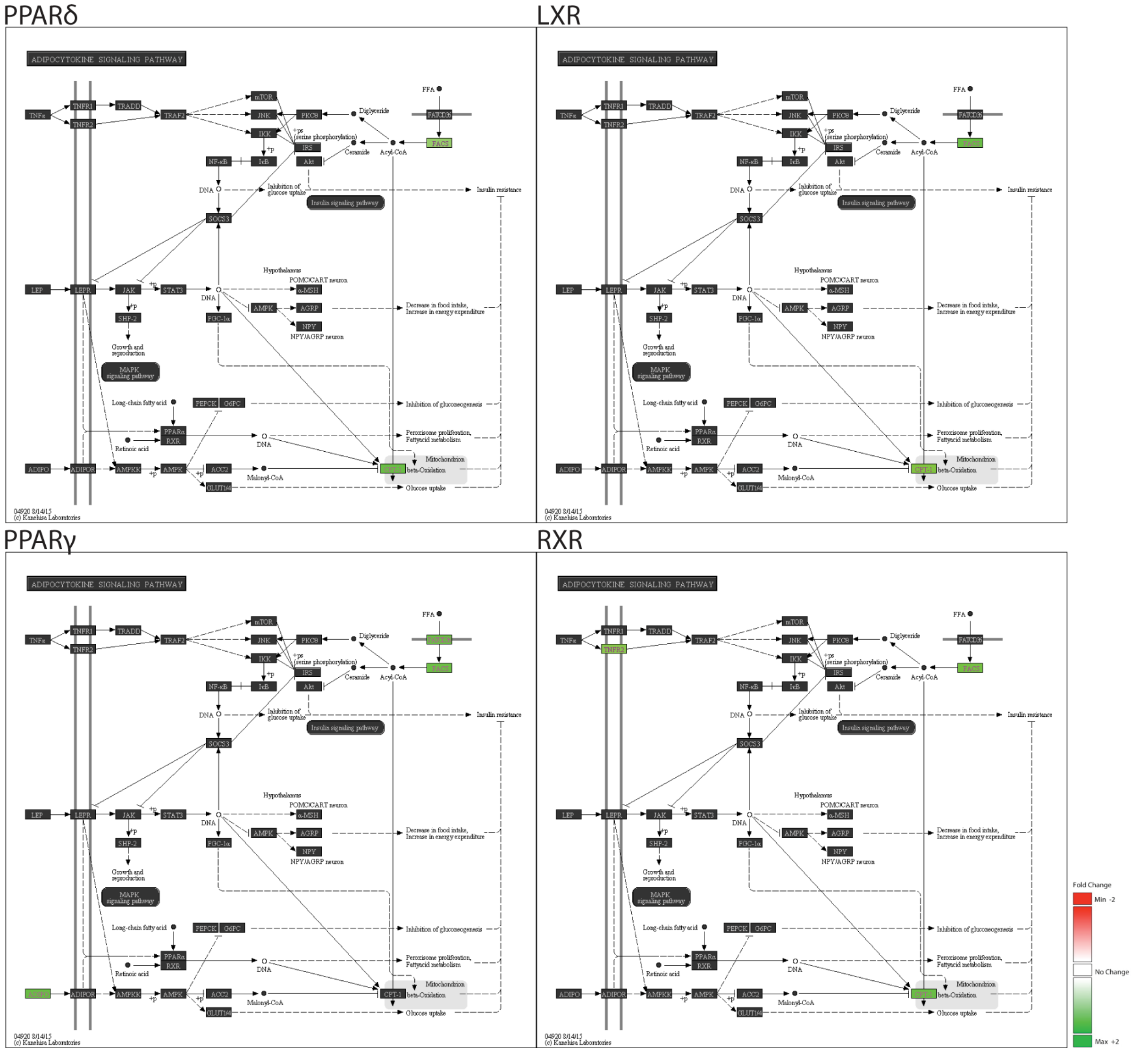


**Supplementary Figure 2: Pathway mapping of adipocytokine signaling.** The adipocytokine signaling pathway is significantly enriched in all four nuclear receptor agonist treatments. Differentially expressed genes as a result of each nuclear receptor agonism is mapped onto the adipocytokine pathway, with gene expression fold-changes ranging from -2 to +2 represented by a color gradient of red or green, respectively.

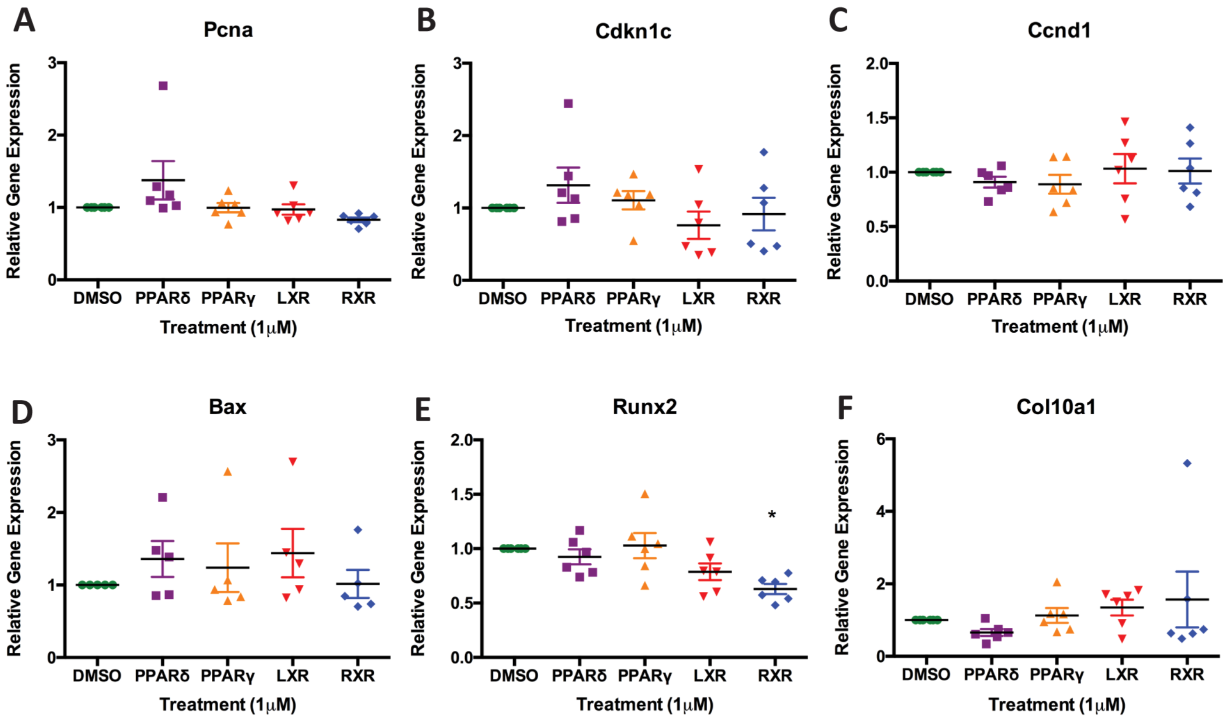


**Supplementary Figure 3: Effects of nuclear receptor agonist treatment on cell proliferation and viability gene expression.** IMACs were incubated for 72 hours with 1μM DMSO (vehicle control), as well as the respective agonists of PPARδ, PPARγ, LXR or RXR. (A, B, C, D, F) Relative gene expression of cell viability markers *Pcna*, *Cdkn1c*, *Ccnd1*, and *Bax*, as well as chondrocyte maturation marker *Col10a1* are not significantly changed with all four nuclear receptor activation. (E) Relative gene expression of terminal chondrocyte differentiation marker *Runx2* is significantly decreased with RXR activation but not by any of the other three nuclear receptor agonists. Values represented are the mean ± SEM of ≥ 3 independent cell isolations. *= $p<0.05$.

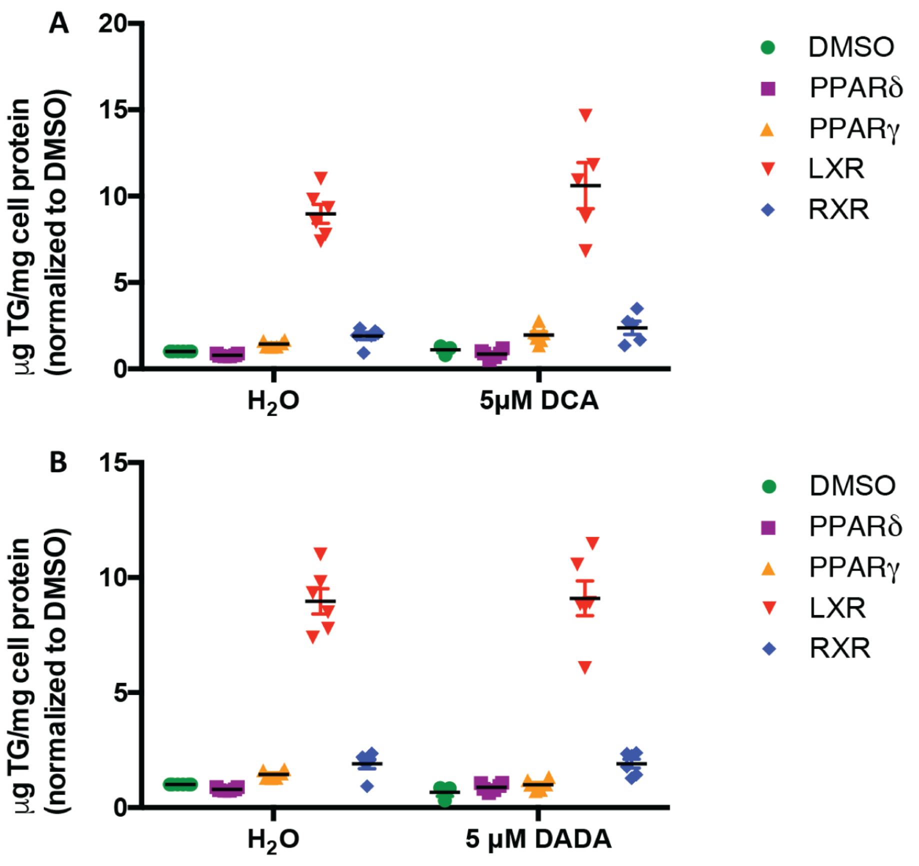


**Supplementary Figure 4: Quantification of cellular lipid mass in nuclear receptor activated IMACs treated with Pdk-inhibitors.** IMACs were incubated for 72 hours with 1µM DMSO (vehicle control), PPARδ agonist GW501516, PPARγ agonist Rosiglitazone, LXR agonist GW3965, or RXR agonist SR11237, together with water (vehicle), DCA or DADA. Lipids were extracted, isolated and mass was measured spectrophotometrically. Proteins were isolated and quantified using BCA. Measurements are reported relative to mg of cell protein. (A) Cell triglycerides (µg) are not significantly altered by dichloroacetic acid (DCA) treatment in PPARγ, PPARδ, LXR, or RXR agonist treated chondrocytes. (B) Levels of cell triglycerides also remain unchanged with diisopropylamine dichloroacetate (DADA) treatment in PPARγ, PPARδ, LXR, or RXR agonist treated chondrocytes. Values represented are the mean ± SEM of ≥ 5 independent cell isolations. *$p<0.05$.
